# Outcomes of an integrated care pathway for concurrent major depressive and alcohol use disorders: a multisite prospective cohort study

**DOI:** 10.1186/s12888-018-1770-3

**Published:** 2018-06-13

**Authors:** Andriy V. Samokhvalov, Charlotte Probst, Saima Awan, Tony P. George, Bernard Le Foll, Peter Voore, Jürgen Rehm

**Affiliations:** 1 Addictions Division, Centre for Addiction and Mental Health (CAMH), 33 Russell Street, Office T519, Toronto, ON M5S 2S1 Canada; 2 Institute for Medical Science, University of Toronto, Toronto, ON Canada; 3 Institute for Mental Health Policy Research, CAMH, Toronto, ON Canada; 4 Division of Brain and Therapeutics, Department of Psychiatry, University of Toronto, Toronto, ON Canada; 5 Campbell Family Mental Health Research Institute, Toronto, ON Canada; 6 Division of Adult Psychiatry and Health Systems, Department of Psychiatry, University of Toronto, Toronto, ON Canada; 7 Dalla Lana School of Public Health, University of Toronto, Toronto, ON Canada; 8 Institute for Clinical Psychology and Psychotherapy, Technische Universität Dresden, Dresden, Sachsen Germany; 9 WHO Collaborating Centre on Mental Health and Addiction, Toronto, ON Canada

**Keywords:** Concurrent disorders, Alcohol use disorder, Major depressive disorder, Care pathway, Pharmacotherapy, Psychotherapy, Integrated treatment

## Abstract

**Background:**

In 2013, an Integrated Care Pathway (ICP) for concurrent Major Depressive (MDD) and Alcohol Use (AUD) Disorders was developed at the Centre for Addiction and Mental Health (CAMH), Toronto, Ontario, Canada. The ICP was further implemented at 8 other clinical sites across Ontario (the DA VINCI Project) in 2015–2017. The goal of this study was to systematically describe and analyze the main clinical outcomes of the project.

**Methods:**

Data on a non-randomized cohort of patients receiving ICP-based treatment were collected prospectively at nine clinical sites in a variety of clinical settings. Statistical methods: descriptive statistics, t-test, chi-square, ANOVA, generalized linear models.

**Results:**

Two hundred forty-six patients were enrolled, 58.8% males, mean age was 45.6 years, 170 patients received treatment at academic health centres (AHC), 49 – at community hospitals (CH) and 27 – in family health teams (FHT). There were no major differences in anamnestic parameters and depression severity between the three settings, but there were differences in baseline drinking patterns between subgroups (F = 4.271, df = 2, *p* = 0.015). Overall completion rate was 70.7% with no significant variation between settings (χ^2^ = 3.35, df = 2, *p* = 0.19). Treatment duration in AHC was the longest, and completion rates were the highest. There was a statistically significant and clinically meaningful reduction in the number of drinking days per week (1.81, *t* = 8.78, *p* < 0.001). The cohort overall demonstrated significant and meaningful reduction in severity of cravings (Penn Alcohol Craving Scale: 4.42, *t* = 8.63, *p* < 0.001) and depressive symptoms (Quick Inventory of Depressive Symptomatology: 4.25, *t* = 11.26, *p* < 0.001). While some of the baseline patient characteristics and treatment parameters varied between the settings, the variation in clinical outcomes was mostly insignificant, though clinical improvement was more pronounced in academic setting and with individual therapy.

**Conclusions:**

The study demonstrated that ICP is a feasible and effective treatment for concurrent AUD and MDD that delivers meaningful clinical improvement in a variety of settings. A randomized controlled study is needed to properly compare the treatment outcomes between ICP model and treatment as usual and to further explore the role of various factors on treatment outcomes.

**Electronic supplementary material:**

The online version of this article (10.1186/s12888-018-1770-3) contains supplementary material, which is available to authorized users.

## Background

In 2013, an Integrated Care Pathway (ICP) for concurrent Major Depressive (MDD) and Alcohol Use (AUD) Disorders was developed at the Centre for Addiction and Mental Health (CAMH), Toronto, Ontario, Canada [1]. These two conditions were chosen as both of them are highly prevalent in Canada and worldwide [2, 3] and often are comorbid with each other, which significantly complicates their effective treatment [4]. Both conditions are also associated with high socioeconomic burden [5, 6] and there is a lack of well-established evidence-based treatments for the treatment of concurrent MDD and AUD [7]. The ICP was created in order to address this systemic shortcoming and after a short pilot stage showed promising clinical results [8, 9]. In 2015 the ICP received support through a joint funding program Adopting Research To Improve Care (ARTIC) in order to implement this ICP at multiple clinical sites across the province of Ontario. The 22-month project was named DA VINCI (Depression and Alcoholism: Validation of an Integrated Care Initiative) and was completed in January 2017 with the ICP fully implemented at nine clinical sites including CAMH.

The goal of this study was to summarize the main clinical outcomes of the implementation of ICP in a variety of clinical settings and to explore the determinants of clinical improvement in patients receiving treatment within the ICP paradigm.

Objectives of the study were to evaluate the treatment completion rate as well as the changes in severity of depressive symptoms, cravings for alcohol and patterns of drinking over the course of treatment.

The primary hypothesis was that patients receiving treatment through the ICP would have a retention rate significantly higher than the retention rate in patients who received treatment as usual (TAU) as previously reported for the central site (CAMH, 42.0 and 30.9% 12- and 16-week retention rates, respectively) [9].

Secondary hypotheses 1–3: There would be a significant reduction in 1) severity of depressive symptoms, 2) severity of cravings, and 3) weekly alcohol consumption levels over the course of ICP treatment.

## Methods

Design: The study is observational in nature and had one prospective cohort of patients receiving ICP treatment at multiple sites. There was no control group. Patients were not randomized into treatment (convenience sample). Data were collected through a review of the clinical charts of all patients enrolled into the ICP since its inception in December 2013 and until September 30th, 2016. The latter time limit was set in order to ensure that all patients had at least 16 weeks to complete the ICP by the time the data collection was completed (January 20th, 2017). No post-treatment follow-up data were collected.

Eligibility criteria: Patients were considered eligible for the ICP when they had concurrent AUD and MDD and were never diagnosed with bipolar disorder. Cases of alcohol-induced depressive disorder were excluded. Patients were diagnosed with alcohol-induced depressive disorder if there was a clear temporal association between the onset of depressive symptoms and alcohol intoxication or withdrawal. Alcohol-induced depressive disorder was ruled out if the onset of depressive symptoms preceded the onset of excessive alcohol use, the symptoms persisted for a substantial period of time after the cessation of alcohol use or there was a clear history of non-alcohol-related depressive episodes as per DSM-5 diagnostic criteria and guidelines for differential diagnosis [10]. There were no age restrictions and patients with a wide variety of sociodemographic and clinical characteristics were included to ensure delivery of treatment to a broader population of eligible patients. Patients who required medical alcohol withdrawal management received it first and then were reassessed and enrolled into the project.

Setting: The project was carried out at 8 sites in Ontario in addition to CAMH, or 9 in total. These sites included 2 academic health centres (AHC): Royal Ottawa Mental Health Centre, Toronto Western Hospital / University Health Network; 3 community hospitals (CH): Trillium Health Partners, North Bay Regional Health Centre, and William Osler Health System and 3 large family health teams (FHT): Hamilton FHT, Village FHT and Inner City FHT.

Intervention: The ICP model includes several crucial components: Pharmacotherapy for both MDD and AUD - treatment was provided by trained physicians who followed the manualized treatment algorithm, which included several antidepressants of different pharmacological classes (sertraline, fluoxetine, venlafaxine and mirtazapine) [11–13] and several medications for the treatment of AUD (naltrexone, acamprosate and topiramate) [7, 12, 14] (See Additional file 1 for details). The algorithm was based on several clinical measures to monitor patients’ therapeutic responses and justify the changes of medications and/or their dosages [15, 16]. These measures included quick inventory of depressive symptomatology (QIDS) [17], Penn alcohol craving scale (PACS) [18] and patterns of drinking operationalized via the following four indicators: number of standard drinks consumed on weekly basis (SD/w), number of drinking days per week (DD/w), number of heavy drinking days per week (HDD/w), and the number of standard drinks consumed per drinking day (SD/DD). Standard drink was defined as any alcoholic beverage containing 13.6 g of ethanol as per the Canada’s Low-Risk Drinking Guidelines [19]. Another important component of the ICP was a 16-week manualized psychotherapy protocol which combined empirically supported treatments for both MDD and AUD such as cognitive behavior therapy (CBT) and motivational interviewing [20]. Initial sessions (1–2) focused on building therapeutic alliance, introduction of the treatment model and motivational enhancement, sessions 3–5 focused on behavioral components and gradually transitioned to cognitive techniques in sessions 6–10. Starting with session 11 protocol focused on relapse prevention, strengthening and rehearsal of the skills acquired during treatment, preparation for transition into community [1, 8]. Psychotherapy was provided weekly by trained clinicians in group or individual format depending on the setting. Also, where possible the ICP was supported by pharmacists and nurses who monitored patients’ medication compliance and provided psychoeducation when necessary. Finally, all clinicians involved in the ICP were meeting on weekly basis to increase the cohesion and integrative collaboration of the clinical team.

Outcome measures:

1. ICP completion rates: the treatment was considered completed if patients had received at least 12 core ICP psychotherapy sessions while being enrolled into the pathway or, in few cases, when they have completed the psychotherapy manual in accelerated pace and demonstrated significant clinical improvement (achieved remission from both MDD and AUD).
2. Severity of depressive symptoms: Measured by 16-item Quick Inventory of Depressive Symptomatology (QIDS-SR_16_ [17]) at baseline and biweekly throughout the treatment.
3. Severity of cravings was measured by Penn Alcohol Cravings Scale (PACS [18]) biweekly.
4. Drinking patterns: Self-reported at each biweekly clinical visit and included number of drinking days per week (DD/w), number of standard drinks per week (SD/w), number of standard drinks per drinking day (SD/d) and number of heavy drinking days (HDD) per week (HDD/w).
5. Severity of depressive symptoms and cravings and drinking patterns were measured at baseline and at the end of treatment (or last clinical visit for patients who dropped out).

Data collection: Data were extracted from clinical charts by three independent raters. Clinical characteristics included medical history, drug use history, alcohol use and AUD history, history of depressive symptoms and MDD, history of treatments for conditions, current drinking patterns and comorbidities. The data from 10 charts were extracted by two raters simultaneously in order to ensure reliable data extraction and inter-rater agreement was evaluated for key variables. Furthermore, data extracted by one rater were randomly checked by other raters for potential extraction errors. In cases of disagreement, the principal investigator reviewed the issue and a consensus on extraction was reached.

Statistical methods: All analyses were performed in R v.3.3.1 [21]. Means and proportions were used to describe the baseline and end-of-treatment characteristics of the sample, which were then compared using paired t-test. ANOVA was used to compare the difference between subgroups of subjects based on the type of clinical settings (AHC, HC, FHT), and type of therapy they received (group or individual). Chi-square test for homogeneity was used for between-group comparisons of categorical variables. A series of linear models were calculated to evaluate the impact of setting and therapy format on clinical outcomes. All tests were bidirectional and based on alpha error of 5%. For non-completers intent-to-treat approach was used [22]. Missing data were imputed using last observation carried forward [23].

Research ethics board: The study received approval from CAMH REB (protocol #053–2016).

## Results

### Patient characteristics

A total of 396 patients with concurrent MDD and AUD were enrolled into ICP across the 9 sites including 44 patients who started their treatment before the DA VINCI project was funded (pilot group) and the 81 patients included into previous report [24]. Out of these, we have selected 246 patients who received treatment within the specified timeframe – 170 patients received treatment in academic health centres (AHC), 49 – in community hospitals (CH) and 27 – in family health teams (FHT). Overall, 58.8% of the samples were men, with the CH subgroup having almost equal distribution of men and women, insignificantly higher proportion of men in AHC and lower – in FHT. Mean age was 45.6 years, there were no significant differences between subgroups (F = 0.364, df = 2, *p* = 0.695). More than half of patients were tobacco smokers (52.4%); majority of them (70.7%) used cannabis in the past month.

On average patients had 14.85 years of AUD history and 12.39 years of MDD history, with no significant difference between settings (F = 0.47, df = 2, *p* = 0.625, and F = 1.22, df = 2, *p* = 0.299, respectively). While severity of depressive symptoms as per QIDS did not vary much between subgroups with the average score of 15.02 (SD = 5.12), the severity of cravings varied insignificantly (17.94, SD = 8.35, F = 2.363, df = 2, *p* = 0.096), and there were significant differences in patterns of drinking (see Table 1). The average number of SD consumed weekly was 34.37 (SD = 35.13), the average number of drinking days per week was 4.00 (SD = 2.96), and the average number of standard drinks per drinking day was 6.24 (SD = 5.16). Overall, the lowest alcohol consumption was reported in patients receiving treatment in community hospitals and the highest in family health teams. The only drinking-related parameter that did not differ significantly between settings was the number of heavy drinking days per week (3.50, SD = 3.07, on average in the whole sample).

**Table 1:** Baseline characteristics of study participants

Characteristic	AHC^a^ (*n* = 169)	CH^b^ (*n* = 49)	FHT^c^ (*n* = 27)	Test statistic	All subjects (*n* = 246)
Sex, %					
Men	63.3%	51.0%	44.4%	χ^2^ = 4.94, df = 2, *p* = 0.08	58.8%
Women	36.7%	49.0%	55.6%		41.2%
Age, mean (SD)	45.42 (11.37)	46.80 (13.83)	44.63 (11.75)	F = 0.364, df = 2, *p* = 0.695	45.61 (11.75)
Smokers, %	51.8%	61.2%	40.7%	χ^2^ = 3.02, df = 2, *p* = 0.22	52.4%
Cannabis users^d^, %	70.6%	77.6%	59.3%	χ^2^ = 2.81, df = 2, *p* = 0.25	70.7%
AUD history, years, mean (SD)	14.29 (11.65)	15.56 (12.60)	16.52 (12.88)	F = 0.47, df = 2, *p* = 0.625	14.85 (11.99)
Drinking patterns:					
SD/w, mean (SD)	37.28 (37.27)	20.96 (26.34)	41.78 (26.86)	F = 4.271, df = 2, *p* = 0.015	34.37 (35.13)
SD/d, mean (SD)	6.59 (5.38)	4.31 (4.46)	7.89 (3.43)	F = 4.509, df = 2, *p* = 0.012	6.24 (5.16)
HDD/w, mean (SD)	3.68 (3.13)	2.60 (2.95)	4.06 (2.57)	F = 2.481, df = 2, *p* = 0.086	3.50 (3.07)
DD/w, mean (SD)	4.11 (2.99)	3.14 (2.92)	5.11 (2.25)	F = 3.325, df = 2, *p* = 0.038	4.00 (2.96)
PACS, mean (SD)	17.73 (8.95)	16.90 (7.10)	21.07 (5.86)	F = 2.363, df = 2, *p* = 0.096	17.94 (8.35)
MDD history, years, mean (SD)	13.47 (11.37)	9.71 (8.51)	11.53 (9.66)	F = 1.22, df = 2, *p* = 0.299	12.39 (10.61)
QIDS, mean (SD)	15.03 (5.23)	14.39 (5.07)	16.41 (4.22)	F = 1.185, df = 2, *p* = 0.307	15.02 (5.12)

^a^*AHC* Academic health centre

^b^*CH* Community hospital

^c^*FHT* Family Health Team

^d^Cannabis users were defined as those who used cannabis at least once in the past year

### Treatment parameters

Average treatment duration varied between 99.31–103.11 days in community settings and 134.09 in AHC, which was significantly longer (F = 3.527, df = 2, *p* = 0.031) than treatment at FHT. There were significant differences between settings in terms of number of physician visits with an average of 7.26 visits across the whole cohort (F = 4.183, df = 2, *p* = 0.016). The largest number was observed in community hospitals, followed by FHT and the lowest in academic settings. There were no significant differences in the number of psychotherapy sessions (F = 1.209, df = 2, *p* = 0.300).

### Clinical outcomes

The data on clinical outcomes are presented in Table 2. One hundred seventy-four (70.7%) of patients completed their treatment, with some insignificant variation between sites (χ^2^ = 3.35, df = 2, *p* = 0.19). On average, there were statistically significant and clinically meaningful reductions in the number of drinking days per week (1.81, *t* = 8.78, *p* < 0.001), number of heavy drinking days per week (2.32, *t* = 10.95, p < 0.001), average number of SD per drinking day (3.23, *t* = 8.65, p < 0.001) and weekly alcohol consumption (22.19, *t* = 9.53, p < 0.001). The drinking patterns changed the most in academic settings with the only parameter significantly varying between settings being DD/w (2.12, F = 3.511, df = 2, *p* = 0.032). The cohort overall demonstrated significant and meaningful reduction in severity of cravings (Reduction in PACS score of 4.42, *t* = 8.63, p < 0.001) and depressive symptoms (reduction of QIDS score of 4.25, *t* = 11.26, *p* < 0.001).

**Table 2:** Summary of the clinical outcomes in the ICP patients

Outcome	AHC^a^ (*n* = 169)	HC^b^ (*n* = 49)	FHT^c^ (*n* = 27)	Test statistic	All subjects (*n* = 246)	Base vs. EoT (paired t-test)^d^
Completion rate, %	74.1%	65.3%	59.3%	χ^2^ = 3.35, df = 2, *p* = 0.19	70.7%	–
Treatment duration, mean (SD)	134.09 (106.51)	99.31 (43.26)	103.11 (30.23)	F = 3.527, df = 2, *p* = 0.031	123.76 (92.36)	
Number of physician visits, mean (SD)	7.65 (4.19)	5.88 (2.64)	6.89 (3.30)	F = 4.183, df = 2, *p* = 0.016	7.26 (3.89)	
Number of therapy sessions, mean (SD)	10.95 (6.03)	12.45 (4.47)	11.41 (6.54)	F = 1.209 df = 2, *p* = 0.300	11.30 (5.82)	
Changes in clinical parameters						
Reduction in SD/w, mean (SD)	25.35 (37.06)	13.39 (26.01)	16.08 (24.03)	F = 2.407, df = 2, *p* = 0.093	22.19 (34.46)	t = 9.53, *p* < 0.001
Reduction in SD/d, mean (SD)	3.57 (6.09)	2.08 (4.05)	3.03 (2.93)	F = 1.251, df = 2, *p* = 0.288	3.23 (5.56)	t = 8.65, *p* < 0.001
Reduction in HDD/w, mean (SD)	2.63 (3.34)	2.06 (2.98)	1.11 (2.64)	F = 2.107, df = 2, *p* = 0.124	2.39 (3.24)	t = 10.95, *p* < 0.001
Reduction in DD/w, mean (SD)	2.12 (3.16)	1.23 (2.85)	0.44 (1.86)	F = 3.511, df = 2, *p* = 0.032	1.81 (3.05)	t = 8.78, *p* < 0.001
Reduction in PACS, mean (SD)	4.57 (8.29)	4.27 (7.38)	3.78 (6.35)	F = 0.128, df = 2, *p* = 0.880	4.42 (7.88)	t = 8.63, *p* < 0.001
Reduction in QIDS, mean (SD)	4.17 (6.22)	4.74 (4.94)	3.77 (3.84)	F = 0.262, df = 2, *p* = 0.77	4.25 (5.76)	t = 11.26, *p* < 0.001

^a^*AHC* Academic health centre

^b^*HC* Community hospital

^c^*FHT* Family Health Team

^d^the difference between the baseline and the end-of-treatment parameters for the whole sample

### Determinants of clinical improvement

Only the reductions in number of SD/w and DD/w were significantly associated with the format of therapy – individual therapy was associated with better outcomes (β = 9.785, 95%CI: 0.371–19.199, *p* = 0.042 and β = 1.051, 95%CI: 0.220–1.882, *p* = 0.013). Correlations between the type of setting and clinical outcomes were in line with the descriptive analyses – treatment in a community hospital and being a patient of FHT was associated with less pronounced reduction in drinking (β = − 11.957, 95%CI: -0.446- -23.468, p = 0.042 and β = − 1.678, 95%CI: -0.199- -1.908, *p* = 0.026, respectively).

## Discussion

The study demonstrated that the ICP paradigm is capable of ensuring high treatment retention rates in complex clinical populations and in a variety of settings. Also, it yielded significant and meaningful clinical improvement in patterns of drinking, severity of cravings and severity of depressive symptoms. Despite the differences in baseline characteristics of patients receiving treatment in the three major types of settings the observed reductions in patterns of drinking, severity of cravings and depressive symptoms were quite similar with slightly better improvements observed in academic settings, likely due to higher availability of clinical resources and higher level of training and specialization of personnel. Also, individual format of therapy and academic settings were associated with significantly better improvement in specific aspects of drinking patterns.

These findings corroborate our preliminary reports on ICP – at the pilot stage, with a small sample or patients (*n* = 28) we reported 80% retention rate at 12 weeks, which is in line with our current definition of ICP completion, and a tendency of reduction in depressive symptoms and alcohol consumption [8]. Later on, in a larger sample of *n* = 81 patients restricted to CAMH only we have demonstrated that ICP patients have significantly higher retention rates in comparison to a convenience sample of matched historical controls (81.5% vs.30.9%) as well as significant and clinically meaningful reduction in depressive symptoms, cravings and alcohol consumption [9]. Thus, with the expansion of the pathway to a variety of settings and despite rather rapid implementation model the ICP still yielded good clinical outcomes comparable to those presented in literature [25].

As the ICP was created and tailored to specific settings and clinical population there are no data on pathways of the same design. At the same time our data are in line with the findings from several randomized clinical trials (RCT) testing effectiveness of several antidepressants and anti-craving medications in depressed alcoholics [11–13, 26]. Out of these the most relevant would be the study of Pettinati and colleagues testing the effectiveness of a combination of naltrexone and sertraline in similar population [12]. Despite less rigorous design and no financial incentives our study yielded retention rates higher than in that RCT (70.7% vs. 57.1%). At the same time the effect sizes for all clinical outcomes in our cohort were significantly smaller than the ones observed in that trial.

In addition to clinical trials of pharmaceuticals there are at least two clinical trials investigating the effects of structured CBT-based clinical pathways [27, 28]. The studies were smaller (*n* = 73 and *n* = 37, respectively), utilized only psychotherapy, but at the same time yielded clinical data comparable to our findings.

In addition, the comparisons to existing literature are complicated due to several methodological limitations of our study, specifically, absence of control group, non-randomized observational design as well as heterogeneity of clinical settings. While the latter aspect of our study allowed to illustrate the impact of the clinical setting on treatment outcomes, the limitations warrant further research aimed to compare the ICP-based treatment to TAU or other treatment models. In addition, the data collection is based on chart review, which though being thorough still leads to a substantial amount of missed data.

Nevertheless, the overall outcomes of the study were positive and corroborate both our previous findings and the results of several studies with similar treatment protocols. Also, as the study is based on a project whose main goal was to broadly implement the ICP protocol in a variety of clinical settings with minimalistic eligibility criteria, we can assume that similar results may be expected in future adaptation and implementation of the ICP protocol in other provinces of Canada as well as internationally.

## Conclusion

The study demonstrated that ICP is a feasible and effective treatment for concurrent AUD and MDD that delivers meaningful clinical improvement in a variety of settings. There was a significant variation in baseline patient characteristics and to some degree – in specific clinical outcomes depending on the setting and format of therapy. Controlled study is needed to properly compare the treatment outcomes between ICP model and treatment as usual as well as to further explore the role of various factors on treatment outcomes.

## Additional file

Additional file 1: The DA VINCI Pharmacotherapy Algorithm. The Supplement contains a description of the DA VINCI Pharmacotherapy Algorithm the way it was presented to all clinical sites. Specifically, the algorithm outlines the medications used for treatment of both AUD and MDD as well as the timelines, rules and clinical scales used to justify the medication dose change or switch to another medication. (PDF 112 kb).

## Abbreviations

AHC: Academic Health Centre
ANOVA: Analysis of Variance
ARTIC: Adopting Research to Improve Care
AUD: Alcohol Use Disorder
CAMH: Centre for Addiction and Mental Health
CBT: Cognitive Behavior Therapy
CH: Community Hospital
DA VINCI: Depression and Alcoholism – Validating an Integrated Care Initiative
DD/w: Number of drinking days per week
FHT: Family Health Team
PACS: Penn Alcohol Craving Scale
QIDS: Quick Inventory of Depressive Symptomatology
QIDS-SR16: Quick Inventory of Depressive Symptomatology – Self-Rated, 16-item
RCT: Randomized Clinical Trial
REB: Research ethics board
SD: Standard deviation
SD/DD: Number of standard drinks per drinking day
SD/w: Number of standard drinks consumed on weekly basis
HDD/w: Number of heavy drinking days per week
ICP: Integrated Care Pathway
MDD: Major Depressive Disorder TAU Treatment as usual
