# Supplementary material for: Outcomes of an integrated care pathway for concurrent major depressive and alcohol use disorders: a multisite prospective cohort study
Source: BMC Psychiatry. 2018 Jun 13;18:189. doi: 10.1186/s12888-018-1770-3 (PMC6001012; doi:10.1186/s12888-018-1770-3)
Supplement: Supplementary file 1 — The DA VINCI Pharmacotherapy Algorithm. The Supplement contains a description of the DA VINCI Pharmacotherapy Algorithm the way it was presented to all clinical sites. Specifically, the algorithm outlines the medications used for treatment of both AUD and MDD as well as the timelines, rules and clinical scales used to justify the medication dose change or switch to another medication. (PDF 112 kb). [file 12888_2018_1770_MOESM1_ESM.pdf]

The DA VINCI Pharmacotherapy Algorithm

DAVINCI
Depression and Alcoholism
Validation of an Integrated Care Initiative

Antidepressant Trials:
Trial A - Sertraline (Fluoxetine, Venlafaxine XR, Mirtazapine -if Sertraline previously tried and failed)
Trial B - Untried drug from Trial A Group
Trial C - Untried drug from Trials A and B Groups

Type	Medication	Dose	Week 1 of medication Day 1	2	3	4	5	6	7	Week 2 of medication 8	9	10	11	12	13	14	Week 3 of medication 15	16	17	18	19	20	21	Week 4 22-28	Week 5 29-35	Week 6 36-42	Week 7 43-49	Week 8 → 50 →	Max Dose
Antidepressants	Sertraline	mg AM	25				50																	Increase dose by 50 mg every 2-4 weeks depending on tolerability and response					200 mg/day
	Fluoxetine	mg AM	10							20														Increase dose by 20 mg every 4 weeks depending on tolerability and response					80 mg/day
	Venlafaxine XR	mg AM	37.5							75														Increase dose by 75 mg every 2-4 weeks depending on tolerability and response					375 mg/day
	Mirtazapine	mg QHS	15							30														Increase dose by 15 mg every 2-4 weeks depending on tolerability and response					60 mg/day

Tactics and Critical Decision Point and Critical Status Plan for the Treatment of Major Depressive Disorder

STARTING POINT → Week 0 (CDP#1) QIDs ≥ 9 Symptomatic PLAN → Initiate antidepressant medication; adjust dose to lower end of therapeutic dose rang or serum level.

QIDS-C16 SCORE	Week 2 of medication	Week 4 of medication	Week 6 of med.	Week 9 of med.	Week 12 of med.
QIDS ≤ 5 (Remission)	Continue current dose.				Go to follow-up phase.
QIDS = 6-8 (Partial Response)	Gradually increase dose as tolerated.	• Continue current dose.<br>• Consider increasing dose.	Increase/maximize dose.	• Increase dose.<br>• Switch to another antidepressant.	• Switch to another antidepressant.<br>• Increase dose and reevaluate in 2 weeks.
QIDS = 6-8 (SEs intolerable)	• Continue current dose and address SEs.<br>• Decrease dose and continue for 2 additional weeks.<br>• Switch to another antidepressant	• Continue current dose and address SEs.<br>• Switch to another antidepressant.	• Continue current dose and address SEs.<br>• Switch to another antidepressant.		
QIDS ≥ 9 (Non-response)	Gradually increase dose as tolerated.	• Increase dose.<br>• Switch to another antidepressant.	Switch to another antidepressant.	Switch to another antidepressant.	Switch to another antidepressant.
QIDS ≥ 9 (SEs intolerable)	• Decrease dose and continue for 2 additional weeks.<br>• Switch to another antidepressant.	• Switch to another antidepressant.			

Type	Medication	Dose	Week 1 of medication Day 1	2	3	4	5	6	7	Week 2 of medication 8	9	10	11	12	13	14	Week 3 of medication 15	16	17	18	19	20	21	Week 4 22-28	Week 5 29-35	Week 6 36-42	Week 7 43-49	Week 8 → 50 →	Max Dose
Anticraving	Trial 1: Naltrexone	mg/day	25				50																						50 mg/day
	Trial 2: Acamprosate	mg TID	333 mg TID	333 mg TID	666 mg TID																								666 mg TID
	Trial 3: Topiramate	mg AM															25							50	50	100	100	150	300 mg/day
		mg PM	25							50															100	100	150	150	

Criteria for Anti-Craving MedicationTreatment Non-Responders	Criteria for Anti-Craving Medication Switch
Treatment non-Responder will have more than 7 (women) or 14 (men) drinks per week on average.<br>OR<br>More than 1 day on which more than 3 drinks (women) or 4 drinks (men) were consumed over the period of 2 weeks.	Being defined as a Treatment non-Responder at 3 consecutive visits will trigger a switch of anticraving medication to the next level.
